# Lipoprotein(a) is associated with DNA damage in patients with heterozygous familial hypercholesterolemia

**DOI:** 10.1038/s41598-024-52571-w

**Published:** 2024-01-31

**Authors:** Ewelina Woźniak, Marlena Broncel, Agnieszka Woźniak, Joanna Satała, Agnieszka Pawlos, Bożena Bukowska, Paulina Gorzelak-Pabiś

**Affiliations:** 1 Laboratory of Tissue Immunopharmacology, Department of Internal Diseases and Clinical Pharmacology, Medical University of Lodz, Lodz, Poland; 2 Department of Biophysics of Environmental Pollution, Faculty of Biology and Environmental Protection, University of Lodz, Lodz, Poland

**Keywords:** Diseases, Cardiovascular diseases, DNA, Lipids, Risk factors

## Abstract

Heterozygous familial hypercholesterolemia (HeFH) is a common autosomal-dominant inherited disorder associated with atherosclerotic cardiovascular disease (ASCVD). HeFH subjects have a higher lipoprotein(a), i.e. Lp(a), concentration than the general population. Patients with FH are exposed to elevated levels of LDL from birth and ox-LDL may induce other oxidation pathways. The aim of the study was to determine the levels of markers of oxidative stress and DNA damage in patients with HeFH and describe the effect of Lp(a) on the resulting damage. Higher DNA damage was identified in patients with HeFH compared to the normolipidemic ones, and ASCVD was associated with greater damage. Oxidative stress markers were elevated in HeFH patients; however, only ox-LDL was higher in the ASCVD group and its level correlated with DNA damage. A positive correlation was found between DNA damage and Lp(a) concentration in the HeFH patients. Higher levels of Lp(a) were associated with greater DNA damage, especially in patients with HeFH and ASCVD. In HeFH patients, the optimal Lp(a) cut-off point associated with ASCVD is > 23.45 nmol/L, i.e. much lower than for the general population; however this cut-off point needs validation in a larger group of HeFH patients.

## Introduction

Heterozygous familial hypercholesterolemia (HeFH) is the most common genetic metabolic disorder, with a prevalence of 1 in 250 individuals, translating into almost 150 million cases worldwide. Individuals with HeFH demonstrate markedly-elevated LDL-C levels from birth, leading to atherosclerotic cardiovascular disease (ASCVD). ASCVD comprises various clinical conditions resulting from atherosclerosis, such as acute coronary syndrome (ACS), myocardial infarction (MI), stable or unstable angina, documented coronary artery disease through angiography, revascularization procedures (e.g., coronary artery bypass graft surgery) stroke, transient ischemic attack, confirmed carotid disease and peripheral artery disease^1^. If untreated, the disease is believed to develop into coronary artery disease (CAD), occurring after age 30 in men and 40 in women. ASCVD is also at least ten times more likely to develop in patients with HeFH. Among this group, the most common cause of death is CAD, with a frequency as high as 60%. HeFH is also associated with cerebrovascular disease (5–10% prevalence)^2^.

Furthermore, HeFH subjects have higher levels of Lp(a), a marker of cardiovascular disease known to induce vascular inflammation, atherogenesis, calcification and thrombosis, compared to the general population^3^. The normal range for Lp(a) is < 75 nmol/L (< 30 mg/dL)^1^; however, an estimated 20% to 25% of the global population have Lp(a) levels > 125 nmol/L (> 50 mg/dL), which the European Atherosclerosis Society associate with increased cardiovascular risk^4^.

Oxidative stress plays an important role in the progression of various cardiovascular diseases^5,6^ and cellular damage, mainly due to lipid peroxidation caused by the action of ROS. Lipid peroxidation can also directly modify DNA^7^. In endothelial cells (ECs), especially in patients with hypercholesterolaemia, the highest amounts of ROS are generated by endothelial nitric oxide synthase (eNOS); these ROS may react with nitric oxide (NO), a vasodilatory compound, thus reducing its bioavailability, and inducing an inflammatory response in the vessel walls^8^. ROS can also increase the susceptibility of lipoproteins to oxidation in damaged vessels by increasing the concentration of oxidized low-density lipoprotein (ox-LDL), which is also associated with a high risk of ASCVD, especially atherosclerosis^9^. In addition, persistent high concentrations of LDL cholesterol may be a source of substrates for oxidation by ROS, leading to increased formation of lipid peroxidation products, and the development of atherosclerosis. Additionally, an excess of LDL-C itself induces oxidative stress by reducing antioxidants and by increasing the activity of enzymes involved in ROS production^8^.

Although patients with FH are exposed to elevated levels of LDL from birth, and ox-LDL may induce other oxidation pathways leading to atherosclerosis and cardiovascular disorders, the level of DNA damage in FH patients has not yet been established. A growing body of clinical and preclinical evidence indicates that DNA damage and the activation of the DNA damage response (DDR) may be associated with a variety of cardiovascular disorders^10–12^. Accumulated DNA damage was shown to be positively correlated with the severity of atherosclerosis in human coronary artery disease^13^.

Among the various genomic insults known to orchestrate a cardiovascular pathology, ROS is a key endogenous influence, known to encourage DNA base oxidation, SSBs, DSBs and telomere shortening^14^. Higher levels of base excision repair (BER) markers have been noted following oxidative DNA damage^12^, suggesting that ROS play a critical role in the accumulation of DNA damage in atherosclerotic lesions. Ox-LDL lowers the level of BER repair enzymes^15^, which are responsible for the removal of 8-oxo-7,8-dihydroguanine (8-OH-Gua) adducts from DNA^16^.

The most commonly-observed DNA lesion induced by oxidative stress is 8-OH-Gua. This has a high potential for mutation, caused by the misincorporation of an adenine instead of cytosine, i.e., a G:C → T:A transversion mutation^17^. Advanced atherosclerotic plaques are characterized by the accumulation of 8-OH-Gua in vascular smooth muscle cells (VSMCs), macrophages, and endothelial cells^18,19^. Shah et al. found 8oxoG repair to be impaired in VSMCs in human atherosclerotic plaques, and attribute this to a reduction in the expression, stability, acetylation and activity of 8oxoG DNA glycosylase I (OGG1) caused by chronic oxidative stress^20^. Such events may contribute to the development of vascular diseases through the accumulation of DNA damage^21^.

Therefore, the aim of the present study is to determine the levels of markers of oxidative stress (ox-LDL, anti-ox-LDL, total antioxidative status), single and double strand-breaks (SSBs, DSBs) in patients with HeFH (with and without ASCVD) and to describe the effect of Lp(a) on the resulting damage.

## Patients and methods

### Subjects with HeFH and controls with normolipidemia

All participants were patients who had been referred to the Department of Internal Diseases and Clinical Pharmacology, Bieganski Memorial Hospital, Lodz for hypercholesterolemia by their primary care physicians. In addition, some patients received a genetic diagnosis of HeFH based on next-generation sequencing (NGS) of FH-related genes, i.e. mutation in the LDL-C receptor (LDLR), apolipoprotein B (APOB) or proprotein convertase subtilisin/kexin type 9 (PCSK9) gene with confirmation by Sanger sequencing. Alternatively, patients who had not been diagnosed with a genetic form of HeFH received a clinical diagnosis of primary hypercholesterolemia, with a clinical diagnosis of HeFH according to the Dutch Lipid Clinic Network (DCLN) Score (i.e. above eight points on the DCLN scale, assessing family history, clinical history, physical examination and LDL-C level)^22,23^

In controls, the main inclusion criteria comprised age 18–65 years with low cardiovascular risk (SCORE2 < 2.5% for patients aged under 50 years, or < 5% for patients aged 50–69 years), LDL-C concentration < 3 mmol/L (< 115 mg/dL), not currently taking any medications, no previous chronic or acute diseases in the past 3 months^24^. In addition, no abnormalities were revealed under physical examination.

The exclusion criteria comprised secondary causes of hypercholesterolemia, including hypothyroidism, kidney diseases, poorly-controlled diabetes, cholestasis or the use of drugs impairing lipid metabolism.

The investigation was approved by the Bioethics Committee of the Medical University of Lodz (RNN/191/21/KE). Informed consent was obtained from all participants. All methods were carried out in accordance with relevant guidelines and regulations.

### Sample collection and diagnostic laboratory methods

All participants were interviewed for their personal history of diabetes, hypertension, smoking, cardiovascular disease, pharmacological treatment, family history of hypercholesterolemia and cardiovascular disease. During the same visit, a physical examination for the presence of corneal arcus and tendon xanthomas was performed.

In both the control and research groups, peripheral blood mononuclear cells (PBMCs) and serum were isolated from peripheral whole blood. All blood samples were obtained after 10 h of fasting. PBMCs were isolated by a centrifugation series using a gradient medium for lymphocyte isolation. The isolated lymphocytes were suspended in a mix (45% RPMI medium, 45% bovine serum and 10% DMSO), then frozen at −80 °C in a box with isopropanol and then stored in liquid nitrogen for up to 28 days.

The lipid profile, viz*.* total cholesterol (TC), LDL, triglycerides (TG), HDL and non-HDL levels, were determined in both cases and controls. The lipid profile was then determined by colorimetric assay. All of the biochemical assays were performed using a Cobas 6000 or 8000 (Roche, Switzerland). All serum or plasma samples intended for the sandwich ELISA analysis were aliquoted and stored at −80 °C for later use. All biochemical measurements were performed in a central laboratory at Bieganski Hospital.

#### Enzyme-linked immunosorbent assay

The serum concentrations of ox-LDL, anti-ox-LDL and total antioxidative status were determined in the control and research groups by sandwich ELISA according to the manufacturer’s instructions (Cloud-Clone Corp., USA).

#### Particle-enhanced immunoturbidimetric assay

The levels of lipoprotein(a) in human serum were quantitatively determined on Roche/Hitachi cobas c systems, using Tina-quant Lipoprotein (a) Gen.2.; LPAM2: ACN 8724. Human lipoprotein (a) was allowed to agglutinate with latex particles coated with anti-Lp(a) antibodies. The precipitate was determined turbidimetrically at 800 / 660 nm. No patient was receiving PCSK9 inhibitor treatment, which could decrease Lp(a) concentration.

#### Comet assay

##### Alkaline version

Damage to DNA was assessed by single-cell gel electrophoresis (comet assay). Briefly, the cells were immersed in low melting point agarose (LMP), placed on microscopic slides, and then lysed; this allowed the released DNA to be subjected to electrophoresis in alkaline conditions (pH > 13). The comet assay enables the identification of SSBs and DSBs, as well as alkali labile sites (ALSs). Slide preparation, electrophoretic separation and staining were conducted according to Woźniak et al.^25^.

##### Comet analysis

The comets were observed at 200× magnification under a fluorescence microscope (Zeiss Axio Scope.A1) connected to an Axiocam 305 color video camera (Carl Zeiss AG, Oberkochen, Germany). The microscope was connected to a desktop PC equipped with Lucia-Comet v. 7.60 software (Laboratory Imaging, Praha, Czech Republic). One hundred images (comets) were randomly selected from each sample and the mean value of DNA in the comet tail was taken as an index of DNA damage (expressed in percent).

#### Statistical analyses

The variables were summarized as median and interquartile range. Categorical data were described using frequencies and percentages. The relationship between categorical variables was assessed using the χ^2^ test. The relationships between groups were tested with the Kruskal–Wallis test (non-normal distribution). Correlations between parameters were assessed by Spearman’s correlation test. The predictive power was evaluated by receiver operating characteristics (ROC) and area under the curve (AUC). The optimal cutoff point in the ROC analysis was chosen with the use of an online tool ‘CutoffFinder’ (https://moLp(a)th.charite.de/cutoff) using ‘Manhattan distance’ (Institut für Pathologie, Charité-Universitätsmedizin Berlin, Berlin, Germany.^9^ Statistical analyses were performed with the GraphPad Prism 9.0 (GraphPad Software, San Diego, California, United States). All tests were considered significant at a *P*-value below 0.05.

## Results

### Clinical characteristics of HeFH and normolipidemia

The study included 73 adult patients (aged 18–65 years). The control group was significantly younger than the patient with HeFH (ASCVD− p = 0,039; ASCVD+ p = 0,0007). No statistically significant differences in age between HeFH patients with and without ASCVD (p = 0,26). There were no significant correlations of age with the parameters we studied (Fig. 3A, B; Supplementary Material 2).

The patients were divided into three groups: (a) control group (n = 20) with normolipidemia and low cardiovascular risk, one study group (b) comprising patients with HeFH but without ASCVD (HeFH ASCVD−) (n = 28), and another group (c) comprising patients with HeFH and ASCVD (HeFH ASCVD+) (n = 25). The ASCVD + patients were those who had experienced one of the following associated with atherosclerosis: ACS, MI, stable or unstable angina, documented coronary artery disease through angiography, revascularization procedures, stroke, transient ischemic attack, confirmed carotid disease or peripheral artery disease^1^. The ASCVD- patients comprised the participants diagnosed with atherosclerosis but who had not experienced any of the above-mentioned incidents. Clinical data, including age, sex, lipid disorders, Lp(a), medical conditions and lipid-lowering treatment, are shown in Table 1.

**Table 1:** Characteristics of the subjects with normolipidemia and HeFH.

Parameter median; IQR	Control, *n* = 20	Total patients with HeFH, *n* = 53	HeFH ASCVD−, *n* = 28	HeFH ASCVD+, *n* = 25	*P* value			
					Control vs. HeFH	Control vs. HeFH ASCVD−	Control vs. HeFH ASCVD+	HeFH ASCVD− vs. HeFH ASCVD+
Sex, n (%)								
Female	17 (85%)	36 (68%)	25 (89%)	11 (44%)	> 0.05	> 0.05	> 0.05	> 0.05
Male	3 (15%)	17 (32%)	3 (11%)	14 (56%)	> 0.05	> 0.05	> 0.05	> 0.05
Age (years)	47.5; 33.75–53	58; 46.25–69.75	52.5; 43–67.75	61; 50–70.5	** < 0.05**	>0.05	** < 0.01**	> 0.05
Medical conditions and hypolipemic therapy, n (%)								
Smokers	0 (0%)	8 (15%)	3 (11%)	5 (20%)	> 0.05	> 0.05	** < 0.05**	> 0.05
Hypertension	2 (10%)	29 (55%)	10 (36%)	19 (76%)	** < 0.05**	> 0.05	** < 0.05**	** < 0.05**
Diabetes type 2	0 (0%)	2 (4%)	0 (0%)	2 (8%)	> 0.05	> 0.05	> 0.05	> 0.05
Prediabetes	0 (0%)	3 (6%)	0 (0%)	3 (12%)	> 0.05	> 0.05	> 0.05	> 0.05
Corneal arcus	0 (0%)	6 (21%)	2 (7%)	4 (16%)	** < 0.05**	> 0.05	> 0.05	> 0.05
Tendon Xanthomas	0 (0%)	6 (21%)	4 (14%)	2 (8%)	** < 0.05**	> 0.05	> 0.05	> 0.05
Untreated	20 (100%)	5 (9%)	5 (18%)	0 (0%)	** < 0.001**	** < 0.001**	** < 0.001**	** < 0.001**
Lipid-lowering therapy								
Low-intensity	0 (0%)	4 (8%)	3 (11%)	1 (4%)	> 0.05	> 0.05	> 0.05	> 0.05
Moderate-intensity	0 (0%)	5 (9%)	1 (4%)	4 (16%)	> 0.05	> 0.05	> 0.05	> 0.05
High-intensity	0 (0%)	39 (74%)	19 (68%)	20 (80%	** < 0.001**	** < 0.001**	** < 0.001**	> 0.05
Laboratory lipid characteristics (mg/dL) and Lpa (nmol/L)								
Total cholesterol	192.5; 163.8–229	215.5;179–283	253; 181–342	209; 161–248	> 0.05	** < 0.01**	> 0.05	> 0.05
Triglycerides	131; 84–194.8	116; 84.3–191.8	115; 76–193	117; 91–202	> 0.05	> 0.05	> 0.05	> 0.05
LDL	112.5; 95.5–137	138.5; 104.3–196.5	174; 107–246	114; 98–168.5	> 0.05	** < 0.01**	> 0.05	> 0.05
HDL	61.7; 48.6–68.3	54; 47.93–65.55	57; 48.4–70.6	53; 44.9–62.7	** < 0.05**	** < 0.01**	> 0.5	> 0.5
Non-HDL	134; 117.5–156.3	164; 123–229.5	200; 125–284	150; 116–189.5	> 0.05	> 0.05	> 0.5	> 0.5
Lpa	10.7; 3.9–23.9	90.7; 12.1–225.3	16.4; 6.8–97.1	199; 46.5–296.5	** < 0.01**	> 0.05	** < 0.001**	** < 0.001**

Significant values are in bold.

The patients were divided into four groups based on the degree of lipid-lowering therapy, viz*.* low (n = 4), moderate (n = 5) and high-intensity (n = 39) and untreated (n = 5)^26–28^. The level of damage was found to be 14.94% (10.36%; 15.73%) in the low group, 19.01% (11.69%; 19.90%) in the moderate group and 14.36% (11.68%; 17.85%) in the high-intensity group and 12.56% (10.11%; 15.35%) in the untreated group. No significant differences in damage level were found with regard to the type of hypolipemic therapy (p = 0.55).

### Among the patients with HeFH, those with ASCVD (HeFH ASCVD+) demonstrated higher levels of DNA damage than those without ASCVD (HeFH ASCVD−)

**Figure 1:**
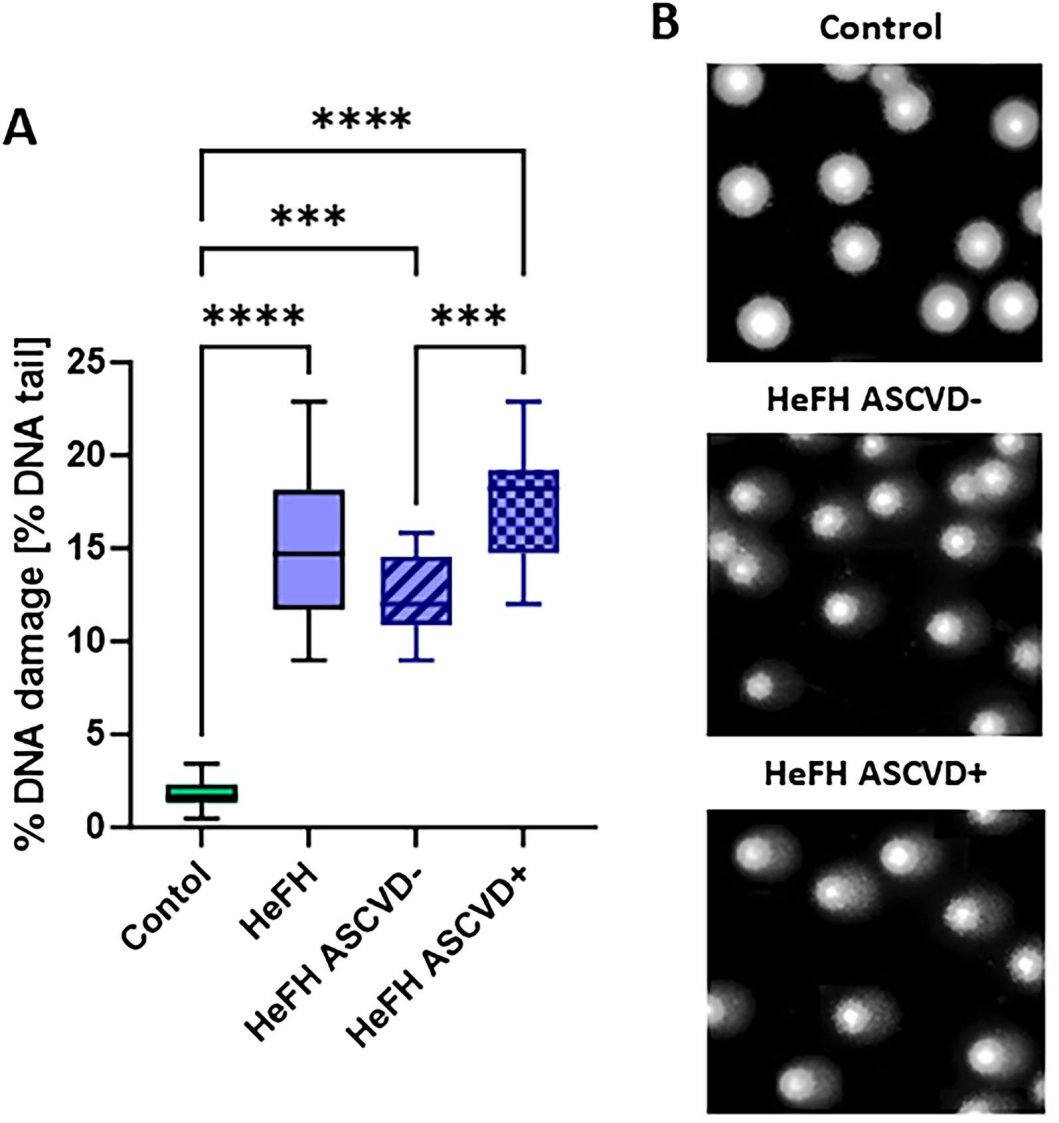
(A) Percentage of DNA in the comet tail from the DNA of patients with normolipidemia (control) and HeFH without and with cardiovascular event (HeFH ASCVD−/HeFH ASCVD+). (**B**) Selected photographs of DNA (comets) of patients. Significant differences are indicted by *p < 0.05; **p < 0.01; ****p < 0.0001.

Significantly higher DNA damage, comprising SSBs, DBSs and alkali labile sites (ALSs), was noted in patients with HeFH compared to those with normolipidemia. The HeFH ASCVD+ patients had higher levels of DNA damage (SSBs, DBSs and ALSs) than the HeFH ASCVD− patients. The percentages of DNA in the comet tail of patients with normolipidemia and HeFH are shown in Fig. 1A, B, together with selected comets.

### The patients with HeFH had higher levels of oxidative stress markers (ox-LDL, anti-ox-LDL, total antioxidative status) than those with normolipidemia

**Figure 2:**
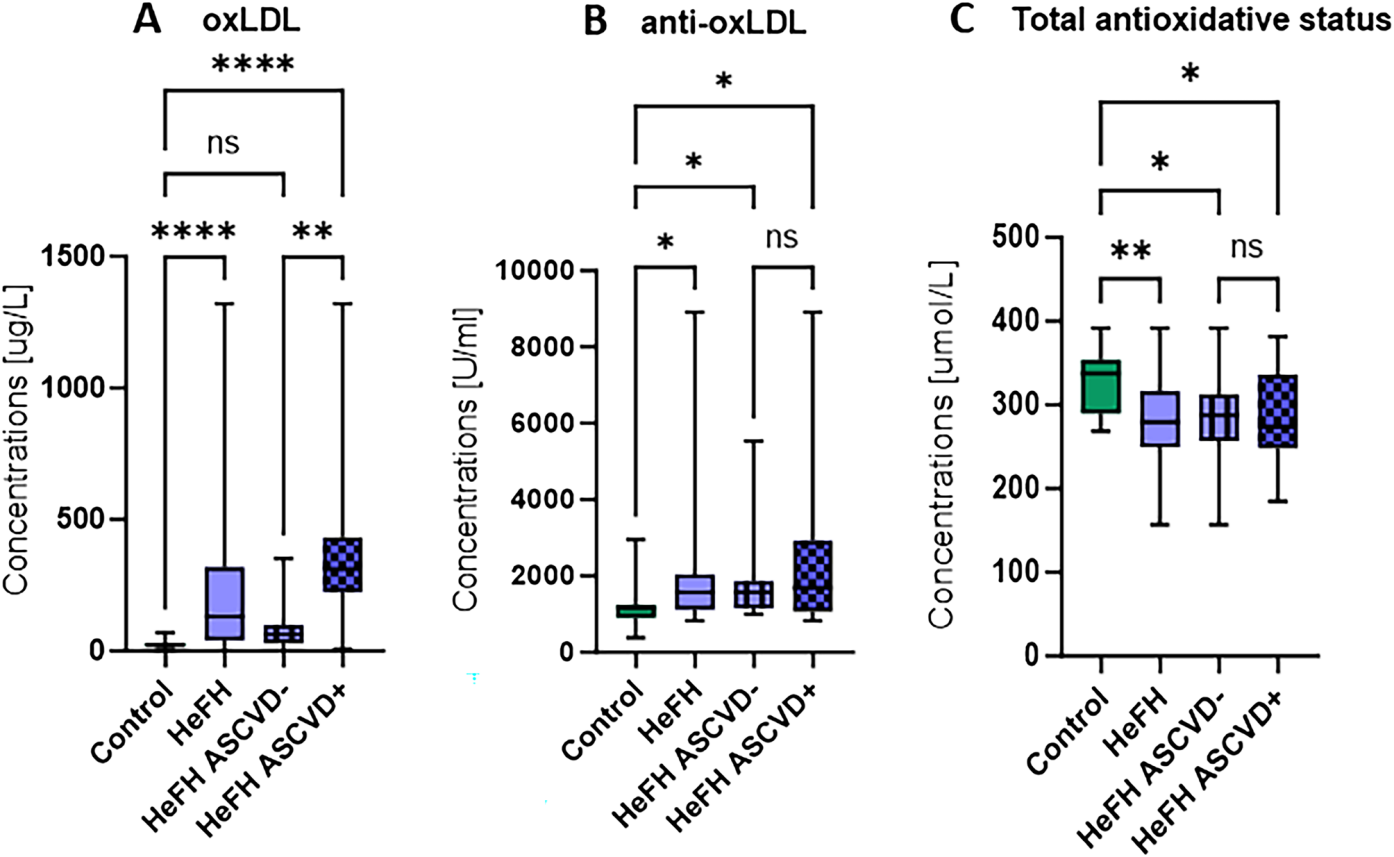
Levels of oxidative stress markers such as (**A**) ox-LDL, (**B**) anti-ox-LDL, and (**C**) total antioxidative status of patients with normolipidemia (control) and HeFH, with and without cardiovascular event (HeFH ASCVD−/HeFH ASCVD+). Significant differences are indicted by **p < 0.01; ***p < 0.001; ****p < 0.0001.

Significantly higher levels of oxidative stress markers such as ox-LDL, anti-ox-LDL and total antioxidative status were identified in patients with HeFH compared to those with normolipidemia (Fig. 2A–C). Amonfg the HeFH patents, those with ASCVD had higher levels of ox-LDL than those without ASCVD. No significant differences were found between these groups with regard to anti-oxLDL or total antioxidative status (Fig. 2A–C).

### The patients with HeFH and ASCVD demonstrated a stronger positive correlation between DNA damage and Lp(a) level than those with HeFH but without ASCVD

**Figure 3:**
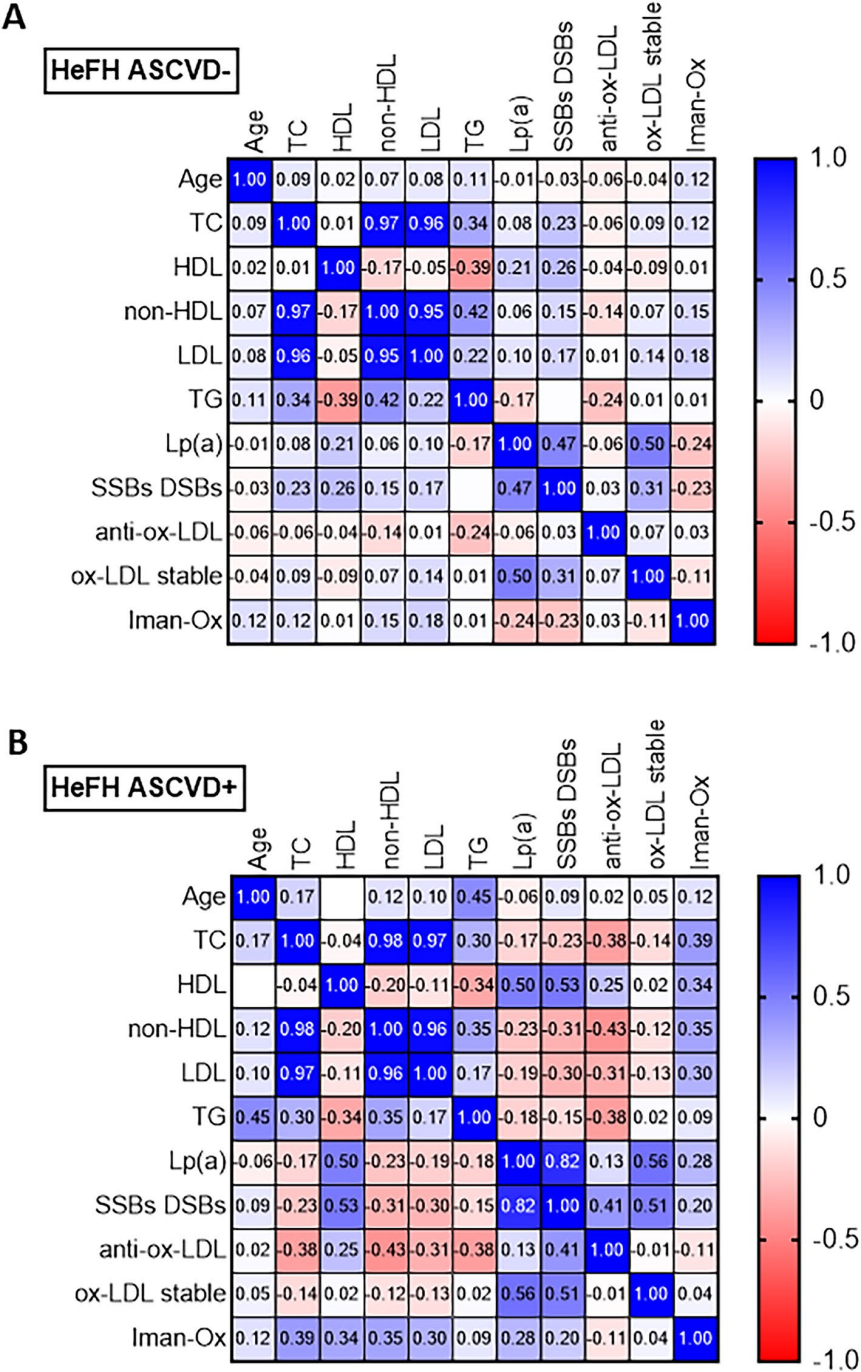
Spearman’s correlation heatmap. Blue squares indicate significant positive correlations (r > 0.5, p < 0.05), white squares indicate non-significant correlations (p > 0.05), and red squares indicate significant negative correlations (r < −0.5, p < 0.05).

The HeFH ASCVD− patients demonstrated a positive correlation between the level of DNA damage and Lp(a) (r = 0.47; p < 0.05) (Fig. 3A). In addition, the HeFH ASCVD+ patients demonstrated a fairly strong positive correlation between DNA damage and Lp(a) (r = 0.82; p < 0.001), and a moderate relationship between DNA damage and ox-LDL (r = 0.51; p < 0.056) (Fig. 3B).

All patients with HeFH, either with or without ASCVD, demonstrated a moderate positive correlation between Lp(a) level and ox-LDL (respectively: r = 0.51 and 0.50, p < 0.05) (Fig. 3A, B).

### The patients with HeFH and ASCVD demonstrated higher Lp(a) levels than those with HeFH but without ASCVD

**Figure 4:**
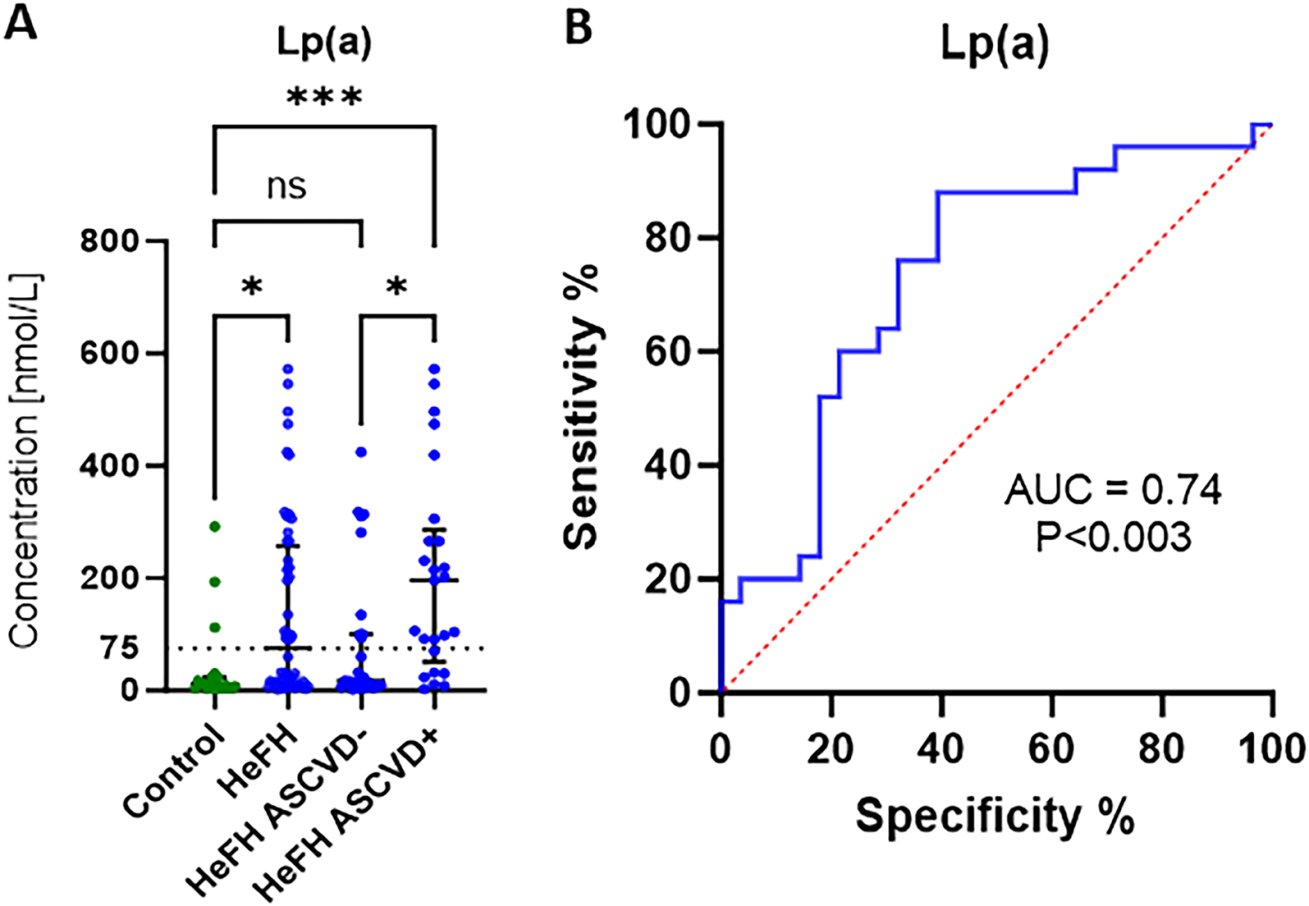
(**A**) Lp(a) level in normolipidemia (control) and HeFH with and without cardiovascular event (HeFH ASCVD−/HeFH ASCVD+) patients. (**B**) Receiver operating characteristic (ROC) curves showing Lp(a) serum level in patients with HeFH.

Significantly higher Lp(a) levels were identified in the HeFH patients compared to those with normolipidemia: 72% (18/25) of HeFH ASCVD+ patients were found to have Lp(a) levels above normal (> 75 nmol/L), compared to 32% (9/28) of HeFH ASCVD− patients. Of the 20 patients with normolipidemia, three (15%) demonstrated elevated Lp(a) levels (Fig. 4A).

### The HeFH patients with ASCVD were found to have an 88% probability of having Lp(a) > 23.45 nmol/L

An ROC curve was plotted for predicting ASCVD in patients with HeFH based on Lp(a) serum levels. The ROC curve for Lp(a) is given in Fig. 4B (AUC 0.74, P < ⁠0.003); the optimal cutoff point (sensitivity 88% and specificity 60.71%) was 23.45 nmol/l. The positive predictive value (PPV) was 88% and negative predictive value (NPV) was 60.7%.

### Lp(a) and DNA damage were found to be associated with ASCVD in HeFH patients.

**Figure 5:**
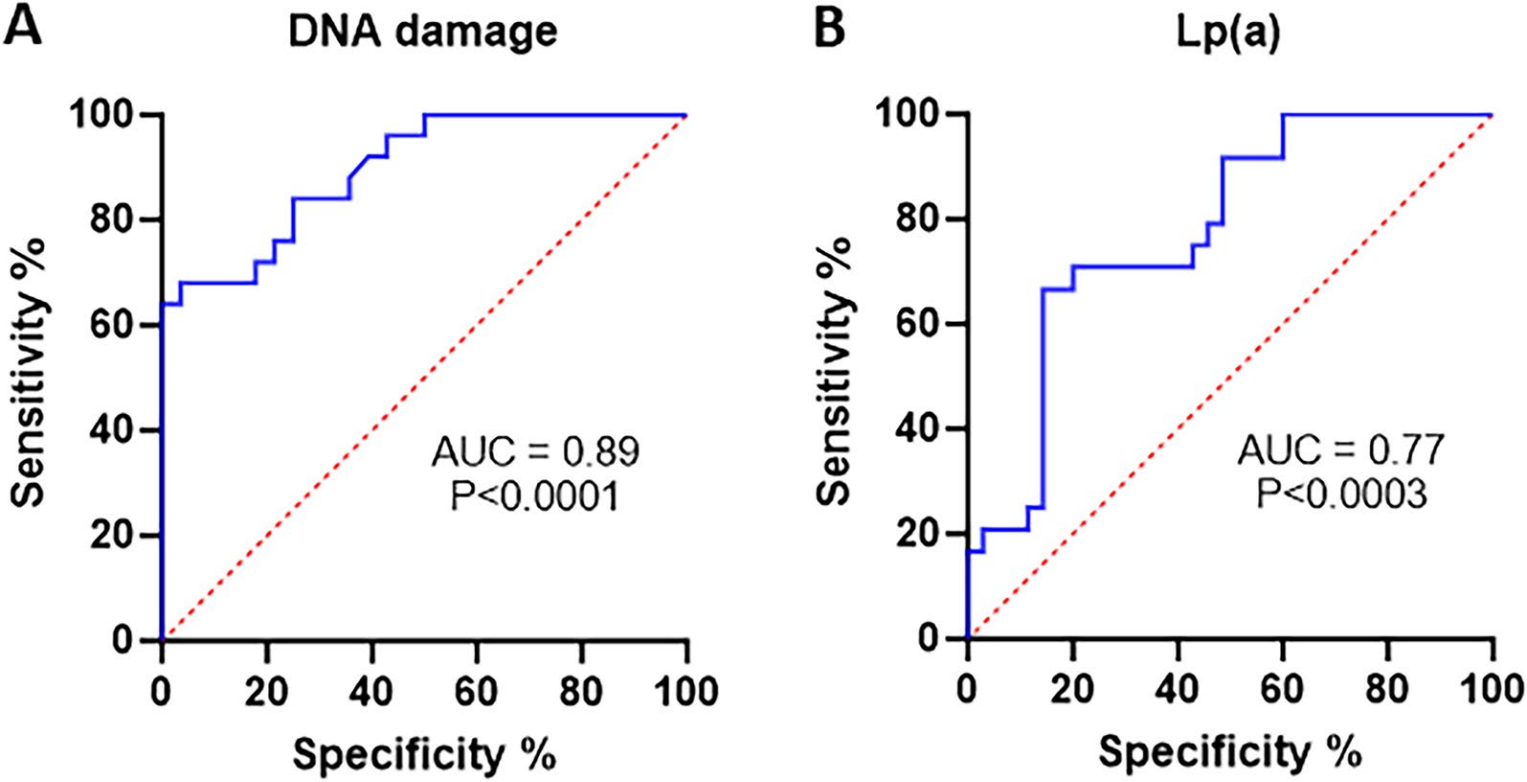
Receiver operating characteristic (ROC) curves showing (**A**) DNA damage and (**B**) Lp(a) serum level in patients with HeFH.

Assuming an area under the ROC curve (AUC) of 0.8964 (p < 0.0001), the optimal DNA damage cut-off point for predicting ASCVD in HeFH with sensitivity 68% and specificity 96.43% was found to be 15.73% (Fig. 5A). Based on this cut-off point, the patients were divided into two groups: (1) patients with DNA damage below the cut-off point (n = 35); (2) patients with DNA damage above the cut-off point (n = 18). PPV was calculated as 68%, and NPV as 96%. The groups differed significantly (p < 0.0001) in Lp(a) concentration: Group 1—17.1 nmol/L (7.9; 92.4), Group 2—225.3 nmol/L (127.7; 432.9).

For this DNA damage cut-off point, based on an AUC value of 0.7786 for the ROC (p < 0.0003), the Lpa level cut-off was found to be 17.8 nmol/L (Fig. 5B). At this cut-off value, the sensitivity of Lpa level in predicting damage in patients with ASCVD and HeFH was found to be 91.47% and specificity 51.43%.

## Discussion

Our present findings suggest that patients with HeFH and ASCVD may be particularly susceptible to DNA damage, and this risk is positively correlated with higher Lp(a) levels. Clinicians should consider assessing Lp(a) level in patients with HeFH, and administering prompt and effective hypolipemic treatment to lower Lp(a) levels and reduce the risk of both ASCVD and DNA damage.

Due to a lack of general awareness of FH among the public and medical community, it is estimated that globally, only 10% of FH population have been accurately diagnosed and adequately treated^29^. It is also estimated that approximately 150,000 of these cases are in Poland^30,31^; however, most of these patients are still unaware of their condition. HeFH is often poorly identified due to the diagnostic limitations associated with genetic testing, and the low level of screening for HeFH. Indeed, cases of FH are often diagnosed only after a premature cardiovascular event^29^.

Although cells are well equipped with DNA damage repair systems, these mechanisms may become overwhelmed or dysfunctional in pathological conditions. As a result, several human diseases including cardiovascular diseases^15^ are characterized by accumulated DNA damage. Patients with hypercholesterolemia demonstrate high plasma LDL concentrations, with LDL-C concentrations two to three times higher than normal; this results in a build-up of LDL in the inner walls of the arteries. This LDL becomes oxidized and promotes the formation of foam cells, thus initiating atherosclerosis. Atherosclerosis is characterized by the presence of elevated markers of DNA damage or replicative stress, such as the phosphorylated histone γH2AX, in endothelial cells lining atheroprone aortic areas (i.e. the aortic arch); these are also noted in macrophages and smooth muscle cells within advanced atherosclerotic lesions in mice and humans^20,32–34^.

Our present findings indicate that patients with HeFH had significantly higher DNA damage than those with normolipidemia. There were no differences in damage levels according to the hypolipemic therapy used. Interestingly, among the patients with HeFH, higher levels of DNA damage were noted in those with ASCVD than in those without. No data currently exists regarding DNA damage in patients with both HeFH and ASCVD, but a growing body of clinical and preclinical evidence associates DNA damage and DDR activation with a variety of cardiovascular disorders^10–12^. DNA damage positively correlates with the severity of ASCAD; in addition, ox-LDL reduces the effectiveness of the enzymes involved in BER-type repair, which exacerbates the development of vascular disease by allowing the accumulation of DNA damage^35^.

Hussein et al. report that cases of FH are characterized by elevated oxidative stress and deficient antioxidative and anti-inflammatory activities in small, dense HDL3. This functional deficiency is closely linked to anomalies in lipid and protein composition, which may prevent HDL from binding to and inactivating oxidized lipids^21^. Similarly, our findings indicate that the patients with HeFH had higher levels of ox-LDL, anti-ox-LDL and lower total antioxidative status compared to normolipidemia patients^36^. However, among the HeFH patients, those with ASCVD demonstrated higher ox-LDL levels, while those without ASCVD did not; no difference was noted in antiox-LDL or total antioxidative status.

Fonzar et al.^37^ report higher levels of the IgM anti-ApoB-D peptide in FH patients with coronary atherosclerosis, and higher IgG anti-OxLDL in those with coronary artery calcium. This suggests the presence of a protective innate response to ApoB and a harmful adaptive response to oxidized LDL. Among patients with HeFH, the level of ox-LDL was found to differ between those with and without ASCVD; however, no statistically significant correlations were observed between ox-LDL level and DNA damage. Interestingly, among all HeFH patients, a positive correlation was observed between Lp(a) and ox-LDL levels. Furthermore, all HeFH patients demonstrated higher Lp(a) levels compared to normolipidemia; this is consistent with Marco-Benedi et al.^3^, who report higher Lp(a) concentrations in subjects with HeFH than in normolipidemia.

Our analysis found the optimal cut-off point for Lp(a) level in HeFH patients associated with ASCVD to be 23.45 nmol/L. An HeFH patient with ASCVD has a 88% probability of having Lp(a) > 23.45 nmol/L, which is three times lower than the normal limit for the general population (< 75 nmol/L). Studies have found patients with HeFH to have a higher risk of ASCVD, and that lowering Lp(a) level by 50 mg/dL (125 nmol/L) for a short period (i.e., five years) reduces ASCVD by 20% in a secondary prevention setting in the general population; hence, there is a need for studies aimed at determining the safe level of Lp(a) for patients with HeFH. This value should be much lower than for the general population^2,38^.

No study has compared the level of DNA damage with the incidence of ASCVD in patients with HeFH. Therefore, one goal of our analysis was to determine the optimal cut-off point for predicting the level of DNA damage indicating ASCVD in HeFH; this point was found to be 15.73%, which was associated with a sensitivity of 68%. Based on this cut-off point, the patients were divided into two groups, i.e. one with less than 15.73% DNA damage and the other with more. Patients who had higher levels of damage also had significantly higher levels of Lp(a) (225.3 nmol/L (127.7; 432.9) vs. 17.1 nmol/L (7.9; 92.4); p < 0.0001). Following this, the cut-off point of Lp(a) level was determined using the area of the ROC curve (0.78, p < 0.0003) this value was found to be 17.8 nmol/L. This cutoff allowed DNA damage to be predicted with a sensitivity of 91.47% in patients with ASCVD and HeFH.

Some reports indicate a positive correlation between LDL and Lp(a)^39^. Marco-Benedi et al.^3^ report that in HeFH, Lp(a) concentration appears to depend on the gene causing FH: mean Lp(a) level was found to be 36.5 mg/dL in APOB-dependent FH but 21.7 mg/dL in LDLR-dependent FH; however, in LDLR-dependent FH, Lp(a) levels did not vary between protein domains, and the higher Lp(a) in HeFH is not explained by higher LDL.

No correlation was found between Lp(a) and LDL levels in the present study. However, it should be remembered that the HeFH patients were treated with lipid-lowering therapy, which reduces LDL levels; as such, no difference in the lipid panel would have been apparent between normolipidemia and HeFH, especially regarding LDL level. Despite receiving maximally-tolerated lipid-lowering therapy (statin plus ezetimibe), the HeFH patients have not achieve their therapeutic goals. For HeFH patients with ASCVD or with at least one risk factor (for example smoking) who are at very-high risk, it is recommended to use treatment aimed at achieving at least a 50% reduction from baseline and an LDL-C < 1.4 mmol/L (< 55 mg/dL). However, no patient was being treated with PCSK9 inhibitors, which could decrease Lp(a) concentration, and this may explain the lack of correlation between LDL and Lp(a) in the HeFH patients in our study. The only difference in the lipid profile between the ASCVD+ and ASCVD- groups was the Lp(a) levels, with the Lp(a) median more than 10 times higher in CVD+ patients.

Our findings indicate a positive correlation between elevated DNA damage and Lp(a) level in all HeFH patients, i.e. those with ASCVD and those without. However, it should be noted that HeFH patients with ASCVD exhibit a stronger correlation (i.e. twice as strong) between DNA damage and Lp(a) level compared to those without ASCVD. As noted above, the lack of significant differences in the lipid panel between HeFH patients with and without ASCVD, together with differences in the level of DNA damage and Lp(a), further indicates that Lp(a) level is associated with higher DNA damage.

No previous studies have found Lp(a) to influence the level of DNA damage; however, in vitro and in vivo studies suggest that oxidized phospholipids (OxPL) on apo(a) demonstrate numerous harmful effects of Lp(a) on monocytes, macrophages, endothelial cells, smooth muscle cells, and valve interstitial cells^40,41^. OxPL are key mediators of the pro-atherosclerotic effects of oxidized lipoproteins and can covalently modify apo(a) or apoB100 proteins or be present in the lipid phase. Both Lp(a) and LDL are apoB-containing lipoproteins, and Lp(a) also contains apo(a). The OxPL content of Lp(a) on apo(a) may explain its potency as a risk factor, despite being present at much lower particle numbers than LDL^42^. This is supported by a recent multivariable analysis in which the inclusion of apoB determinants did not affect the association between genetically-determined Lp(a) levels and coronary artery disease, while no association was found between genetically-elevated LDL-C and coronary artery disease.

## Conclusions

HeFH patients experience DNA damage, elevated oxLDL and anti-oxLDL, and lower total plasma antioxidant capacity compared to patients with normolipidemia. Higher levels of Lp(a) are associated with higher levels of DNA damage, especially in patients with a history of a cardiovascular event. Our analysis indicates the optimal Lp(a) cut-off point for prognosing ASCVD in HeFH patients to be > 23.45 nmol/L Lp(a), which is much lower than for the general population. However, this cut-off point requires further validation in a larger group of HeFH patients.

### Supplementary Information

Supplementary Information 1.

Supplementary Information 2.

Supplementary Legends.

## Data Availability

The raw data send in Supplementary materials.

## References

[1] Pearson GJ, Thanassoulis G, Anderson TJ, Barry AR, Couture P, Dayan N (2021). 2021 Canadian Cardiovascular Society Guidelines for the management of dyslipidemia for the prevention of cardiovascular disease in adults. Can. J. Cardiol..

[2] Nicholls SJ, Tang WHW, Scoffone H, Brennan DM, Hartiala J, Allayee H (2010). Lipoprotein(a) levels and long-term cardiovascular risk in the contemporary era of statin therapy. J. Lipid Res..

[3] Marco-Benedí V, Cenarro A, Laclaustra M, Larrea-Sebal A, Jarauta E, Lamiquiz-Moneo I (2022). Lipoprotein(a) in hereditary hypercholesterolemia: Influence of the genetic cause, defective gene and type of mutation. Atherosclerosis.

[4] Duarte Lau F, Giugliano RP (2022). Lipoprotein(a) and its significance in cardiovascular disease: A review. JAMA Cardiol..

[5] Peoples JN, Saraf A, Ghazal N, Pham TT, Kwong JQ (2019). Mitochondrial dysfunction and oxidative stress in heart disease. Exp. Mol. Med..

[6] Kattoor AJ, Pothineni NVK, Palagiri D, Mehta JL (2017). Oxidative stress in atherosclerosis. Curr. Atheroscler. Rep..

[7] Marnett LJ (2000). Oxyradicals and DNA damage. Carcinogenesis.

[8] Ganjali S, Keshavarz R, Hosseini S, Mansouri A, Mannarino MR, Pirro M (2021). Evaluation of oxidative stress status in familial hypercholesterolemia. J. Clin. Med..

[9] Tsimikas S (2006). Oxidized low-density lipoprotein biomarkers in atherosclerosis. Curr. Atheroscler. Rep..

[10] Matsuoka S, Ballif BA, Smogorzewska A, McDonald ER, Hurov KE, Luo J (2007). ATM and ATR substrate analysis reveals extensive protein networks responsive to DNA damage. Science (80-).

[11] Sano M, Minamino T, Toko H, Miyauchi H, Orimo M, Qin Y (2007). p53-induced inhibition of Hif-1 causes cardiac dysfunction during pressure overload. Nature.

[12] Shukla PC, Singh KK, Quan A, Al-Omran M, Teoh H, Lovren F (2011). BRCA1 is an essential regulator of heart function and survival following myocardial infarction. Nat. Commun..

[13] Pilié PG, Tang C, Mills GB, Yap TA (2019). State-of-the-art strategies for targeting the DNA damage response in cancer. Nat. Rev. Clin. Oncol..

[14] Ait-Aissa K, Blaszak SC, Beutner G, Tsaih SW, Morgan G, Santos JH (2019). Mitochondrial oxidative phosphorylation defect in the heart of subjects with coronary artery disease. Sci. Rep..

[15] Jackson SP, Bartek J (2009). The DNA-damage response in human biology and disease. Nature.

[16] Ciccia A, Elledge SJ (2010). The DNA damage response: Making it safe to play with knives. Mol. Cell..

[17] Basu S, Je G, Kim YS (2015). Transcriptional mutagenesis by 8-oxodG in α-synuclein aggregation and the pathogenesis of Parkinson’s disease. Exp. Mol. Med..

[18] Martinet W, Knaapen MWM, De Meyer GRY, Herman AG, Kockx MM (2001). Oxidative DNA damage and repair in experimental atherosclerosis are reversed by dietary lipid lowering. Circ. Res..

[19] Martinet W, Knaapen MWM, De Meyer GRY, Herman AG, Kockx MM (2002). Elevated levels of oxidative DNA damage and DNA repair enzymes in human atherosclerotic plaques. Circulation.

[20] Shah A, Gray K, Figg N, Finigan A, Starks L, Bennett M (2018). Defective base excision repair of oxidative DNA damage in vascular smooth muscle cells promotes atherosclerosis. Circulation.

[21] Hussein H, Saheb S, Couturier M, Atassi M, Orsoni A, Carrié A (2016). Small, dense high-density lipoprotein 3 particles exhibit defective antioxidative and anti-inflammatory function in familial hypercholesterolemia: Partial correction by low-density lipoprotein apheresis. J. Clin. Lipidol..

[22] Defesche J (2001). World Health Organisation report on familial hypercholesterolemia. Atherosclerosis.

[23] Catapano AL, Graham I, De Backer G, Wiklund O, John Chapman M, Drexel H (2016). 2016 ESC/EAS guidelines for the management of dyslipidaemias. Eur. Heart J..

[24] Visseren F, Mach F, Smulders YM, Carballo D, Koskinas KC, Bäck M (2021). 2021 ESC Guidelines on cardiovascular disease prevention in clinical practice. Eur. Heart J..

[25] Woźniak E, Broncel M, Bukowska B, Gorzelak-Pabiś P (2020). The protective effect of dabigatran and rivaroxaban on DNA oxidative changes in a model of vascular endothelial damage with oxidized cholesterol. Int. J. Mol. Sci..

[26] Bays HE, Patel MD, Mavros P, Ramey DR, Tomassini JE, Tershakovec AM (2017). Real-world data to assess changes in low-density lipoprotein cholesterol and predicted cardiovascular risk after ezetimibe discontinuation post reporting of the Ezetimibe and Simvastatin in Hypercholesterolemia Enhances Atherosclerosis Regression trial. J. Clin. Lipidol..

[27] Compared LA, Intensified W, Monotherapy S (2013). Effectiveness of combination therapy with statin and another lipid-modifying agent compared with intensified statin monotherapy. Ann. Intern. Med. Rev..

[28] Lee J, Egolum U, Parihar H, Cooley M, Ling H (2021). Effect of Ezetimibe added to high-intensity statin therapy on low-density lipoprotein cholesterol levels: A meta-analysis. Cardiol. Res..

[29] Lale T, Meral K (2021). Familial hypercholesterolemia: Global burden and approaches. Curr. Cardiol. Rep..

[30] Dyrbuś K, Gąsior M, Desperak P, Osadnik T, Nowak J, Banach M (2019). The prevalence and management of familial hypercholesterolemia in patients with acute coronary syndrome in the Polish tertiary centre: Results from the TERCET registry with 19,781 individuals. Atherosclerosis.

[31] Banach M, Jankowski P, Joíwiak J, Cybulska B, Windak A, Guzik T (2017). PoLA/CFPiP/PCS guidelines for the management of dyslipidaemias for family physicians 2016. Arch. Med. Sci..

[32] Natarelli L, Geißler C, Csaba G, Wei Y, Zhu M, Di Francesco A (2018). MIR-103 promotes endothelial maladaptation by targeting lncWDR59. Nat. Commun..

[33] Shah NR, Mahmoudi M (2015). The role of DNA damage and repair in atherosclerosis: A review. J. Mol. Cell Cardiol..

[34] Fidler TP, Xue C, Yalcinkaya M, Hardaway B, Abramowicz S, Xiao T (2021). The AIM2 inflammasome exacerbates atherosclerosis in clonal haematopoiesis. Nature.

[35] Khanna KK, Jackson SP (2001). DNA double-strand breaks: Signaling, repair and the cancer connection. Nat. Genet..

[36] Sato K, Yao T, Fujimura T, Murayama K, Okumura K, Hagiwara N (2021). Oxidative stress-responsive apoptosis-inducing protein in patients with heterozygous familial hypercholesterolemia. Heart Vessels.

[37] Fonzar WT, Fonseca FA, Fonseca HA, Silva TP, Rodrigues AA, Teixeira D (2022). Atherosclerosis severity in patients with familial hypercholesterolemia: The role of T and B lymphocytes. Atheroscler. Plus.

[38] Madsen CM, Kamstrup PR, Langsted A, Varbo A, Nordestgaard BG (2020). Lipoprotein(a)-lowering by 50 mg/dL (105 nmol/L) may be needed to reduce cardiovascular disease 20% in secondary prevention: A population-based study. Arterioscler. Thromb. Vasc. Biol..

[39] Zhu L, Zheng J, Gao B, Jin X, He Y, Zhou L (2022). The correlation between lipoprotein(a) elevations and the risk of recurrent cardiovascular events in CAD patients with different LDL-C levels. BMC Cardiovasc. Disord..

[40] Scipione CA, Sayegh SE, Romagnuolo R, Tsimikas S, Marcovina SM, Boffa MB (2015). Mechanistic insights into Lp(a)-induced IL-8 expression: A role for oxidized phospholipid modification of apo(a ). J. Lipid Res..

[41] Van Der Valk FM, Bekkering S, Kroon J, Yeang C, Van Den Bossche J, Van Buul JD (2016). Oxidized phospholipids on Lipoprotein(a) elicit arterial wall inflammation and an inflammatory monocyte response in humans. Circulation.

[42] Marlys LK, Michael BB (2022). Oxidized phospholipid modification of lipoprotein(a): Epidemiology, biochemistry and pathophysiology. Atherosclerosis.

