# Supplementary figures and images for: Lipoprotein(a) is associated with DNA damage in patients with heterozygous familial hypercholesterolemia
Source: Sci Rep. 2024 Jan 31;14:2564. doi: 10.1038/s41598-024-52571-w (PMC10830471; doi:10.1038/s41598-024-52571-w)

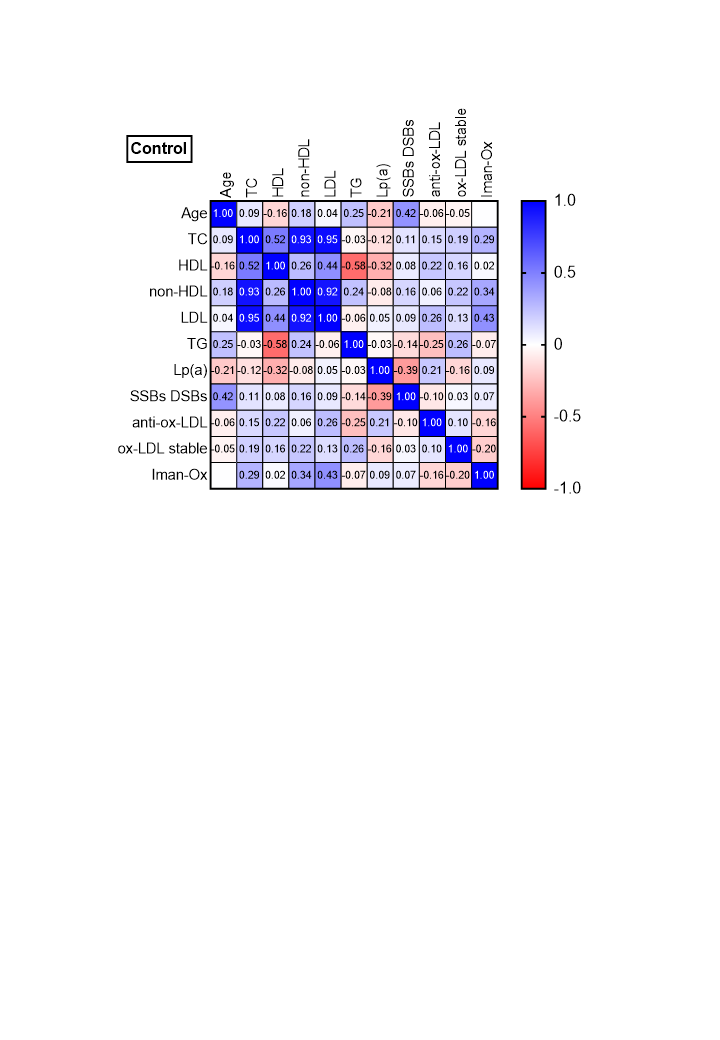

Supplement: Supplementary file 2 — Supplementary Information 2. [file 41598_2024_52571_MOESM2_ESM.tif]
