# Supplementary material for: Lipoprotein(a) is associated with DNA damage in patients with heterozygous familial hypercholesterolemia
Source: Sci Rep. 2024 Jan 31;14:2564. doi: 10.1038/s41598-024-52571-w (PMC10830471; doi:10.1038/s41598-024-52571-w)
Supplement: Supplementary file 3 — Supplementary Legends. [file 41598_2024_52571_MOESM3_ESM.docx]

Supplementary materials 2.   Spearman’s correlation heatmap. Blue squares indicate significant positive correlations (r > 0.5, p < 0.05), white squares indicate non-significant correlations (p > 0.05), and red squares indicate significant negative correlations (r < -0.5, p < 0.05).
